# Mouse Social Network Dynamics and Community Structure are Associated with Plasticity-Related Brain Gene Expression

**DOI:** 10.3389/fnbeh.2016.00152

**Published:** 2016-08-04

**Authors:** Cait M. Williamson, Becca Franks, James P. Curley

**Affiliations:** ^1^Department of Psychology, Columbia University, New York, NYUSA; ^2^Animal Welfare Program, Land and Food Systems, University of British Columbia, Vancouver, BCCanada; ^3^Center for Integrative Animal Behavior, Columbia University, New York, NYUSA

**Keywords:** social networks, community structure, social behavior, social dynamics, social neuroscience

## Abstract

Laboratory studies of social behavior have typically focused on dyadic interactions occurring within a limited spatiotemporal context. However, this strategy prevents analyses of the dynamics of group social behavior and constrains identification of the biological pathways mediating individual differences in behavior. In the current study, we aimed to identify the spatiotemporal dynamics and hierarchical organization of a large social network of male mice. We also sought to determine if standard assays of social and exploratory behavior are predictive of social behavior in this social network and whether individual network position was associated with the mRNA expression of two plasticity-related genes, DNA methyltransferase 1 and 3a. Mice were observed to form a hierarchically organized social network and self-organized into two separate social network communities. Members of both communities exhibited distinct patterns of socio-spatial organization within the vivaria that was not limited to only agonistic interactions. We further established that exploratory and social behaviors in standard behavioral assays conducted prior to placing the mice into the large group was predictive of initial network position and behavior but were not associated with final social network position. Finally, we determined that social network position is associated with variation in mRNA levels of two neural plasticity genes, DNMT1 and DNMT3a, in the hippocampus but not the mPOA. This work demonstrates the importance of understanding the role of social context and complex social dynamics in determining the relationship between individual differences in social behavior and brain gene expression.

## Introduction

Laboratory studies of mouse social behavior typically involve observations of dyadic interactions of non-familiar social partners in a novel environment (Brodkin, 2007; Kas et al., 2014). While these tests provide some basic information on the behavior of a laboratory mouse, there is increasing concern that these tests do not provide sufficient insight into more complex social behaviors such as social competence that may be relevant for translational research (Hofmann et al., 2014; Peters et al., 2015). A critical issue to be resolved is what complex, ethologically relevant social behaviors are laboratory mice able to exhibit? It is known from field studies that the ancestors of laboratory mice (*Mus musculus*) live in large social groups with a high degree of spatial organization (Berry, 1970; Crowcroft, 1973). Additionally, previous studies have shown that both wild mice and laboratory mice in semi-natural environments form territories each with dominant mice that patrol and defend resources such as food or females (Mackintosh, 1970; Mondragón et al., 1987; Gray et al., 2000; Perony et al., 2012). More recently, studies using automated tracking technologies to look at the behavior of laboratory mice living in large groups have revealed similar patterns of spatial and temporal organization, suggesting it is feasible to study such social behavior in the laboratory (Perony et al., 2012; Freund et al., 2013, 2015; Ohayon et al., 2013; Weissbrod et al., 2013; Thanos et al., 2015).

One of the most well-understood types of social organization is the dominance hierarchy, which has been studied in many different species, including insects (Röseler et al., 1984), fish (Maruska and Fernald, 2011), primates (Enger et al., 1954; Machado and Bachevalier, 2006; Noonan et al., 2014), and humans (Kumaran et al., 2012). These hierarchies may be determined through competitive dominance where animals out-compete each other for access to resources or agonistic dominance where animals are judged to be dominant based upon wins and losses against each other during agonistic contests (De Waal, 1989). They may also be represented by formal dominance whereby individuals express behaviors that communicate dominance or subordinate behavior without engaging in fighting (De Waal, 1989). In the wild, social rank in a dominance hierarchy is primarily determined by an individual’s ability to monopolize resources (e.g., food, space, mates) and higher rank is strongly associated with improved reproductive success and fitness (Mooney et al., 2014; Franz et al., 2015). In the laboratory mouse, the majority of social dominance studies have focused on social rank acquisition in dyads or a small number of competing individuals (Curley, 2011). It has also been shown that male mice may form elementary linear dominance hierarchies when animals are repeatedly tested against each other in pairs (Wang et al., 2011; van den Berg et al., 2015). We have previously shown that groups of twelve male mice living together in an ethologically relevant visible burrow system form stable linear dominance hierarchies based upon their expressions of agonistic and formal dominance (So et al., 2015; Williamson et al., 2016).

The aim of the present study was to determine whether male mice living in a large social group of thirty individuals would form a dominance hierarchy. It is not yet known if individuals would be able to hierarchically organize themselves in larger groups which would potentially require greater social learning and competence by all individuals (So et al., 2015; Curley, 2016b; Williamson et al., 2016). Additionally, using statistical modeling and social network analysis, we aimed to identify more complex spatiotemporal patterns of social interactions between individuals, particularly whether individuals would preferentially associate into sub-communities within the larger network. A further aim was to determine whether individual differences in standard tests of social and exploratory behavior were predictive of the social behavior of individuals when living in large stable social groups. Previous studies in a number of species have reported positive and negative associations between personality types such as boldness, exploration and sociability, and dominance rank (Verbeek et al., 1999; Carere et al., 2005; Boogert et al., 2006; Fox et al., 2009; David et al., 2011), and that animals spatially organize themselves according to similarities and dissimilarities in these personalities (Pike et al., 2008; Croft et al., 2009; Aplin et al., 2013; Massen and Koski, 2014; Carter et al., 2015). Thirdly, we examine whether individual differences in social network position are related to individual differences in gene expression of two markers of brain plasticity, DNA (cytosine-5)-methyltransferase 1 (DNMT1), and DNA (cytosine-5)-methyltransferase 3 alpha (DNMT3a), in the hippocampus and medial preoptic area of the hypothalamus (mPOA). While DNMT1 is primarily known to mediate the maintenance of DNA methylation patterns established in early development, this enzyme may also play a role in DNA methylation in post-mitotic neuronal cells and therefore mediate brain plasticity (Champagne, 2010; Jensen Peña et al., 2012). DNMT3a mediates *de novo* methylation patterning and is required for synaptic plasticity, learning and memory (Feng et al., 2010). Indeed, changes in the expression of DNMTs have been associated with behavioral plasticity including learning and memory processes (Miller and Sweatt, 2007; Feng et al., 2010; Yu et al., 2011). Establishing and maintaining position within a social network requires individuals to learn about their relationships with multiple other individuals and to be able to express socially contextual appropriate behavior to all other individuals within their social network (Fernald, 2014). Acquiring such social information and responding to changes in social context has been shown to be associated with a suite of neuroplastic changes in animals of different social status across species (Taborsky and Oliveira, 2012; Cardoso et al., 2015; Fernald, 2015). Further, manipulations of DNMT-dependent DNA methylation has also been shown to lead to changes in social status. In African cichlid fish, upregulating DNA methylation through L-methionine administration leads individuals to become socially dominant, while inhibition of DNMT activity through zebularine administration prohibits individuals from becoming dominant (Lenkov et al., 2015). Silencing DNMT3a through RNA interference in honeybees leads to increased development of queen versus worker bees (Kucharski et al., 2008). Given the role of DNMTs in modulating neural plasticity, learning, memory, and social status and the importance of these mechanisms in regulating both the formation of social hierarchies and the maintenance of socially competent behavior, we hypothesized that changes in the expression of DNMT1 and DNMT3a in two brain regions associated with social behavior and learning and memory would be associated with an individual’s ability to maintain a central social network position.

## Materials and Methods

### Subjects and Housing

Male outbred CD1 mice (*N* = 60) aged 7 weeks were purchased from Charles River and housed in standard sized cages (27 cm × 17 cm × 12 cm) with pine shaving bedding in groups of three for 10 days prior to the start of behavioral testing and throughout the behavioral testing period. Each male placed in the vivarium (1-2 individuals selected randomly from each cage) was given a unique ID (1-30) and distinctively marked with a blue, non-toxic, non-hazardous marker (Stoelting Co.). These marks remain for up to 12 weeks enabling each animal to be clearly identified throughout the study. These 30 males were used as subject animals in the study (**Table 1**). The remaining 30 animals were only used in this study as stimulus animals in social tests. Standard behavioral testing took place over a 15 day period, 3 days after which subject mice were all weighed and randomly placed into one of four custom built mouse vivaria (length 150 cm, height 80 cm, width 80 cm; Mid-Atlantic) (Supplementary Figure S1), which were inter-connected by tubes such that mice could move from one vivarium to another. Each vivarium consisted of three sides of Plexiglas with sliding front doors and a metal backboard containing multiple holes for air circulation. Standard food chow and water was provided *ad libitum* at the top shelf via cage lids that protruded through the vivarium roof. Animals could access each shelf via a system of ramps and tunnels that connected each shelf and side. These same types of tunnels connected each vivarium to the one next to it. Multiple enrichment objects such as plastic igloos and wooden blocks were also provided. The floors of each vivarium were covered with pine shaving bedding. The floors of each vivarium were covered with pine shaving bedding. Bedding was not changed during the vivarium observation period to avoid disturbing mice and interfering with the group structure. Sufficient clean bedding was provided at the beginning of observations in all burrows and shelves that animals could nestbuild with and use throughout observations. The animals were kept in a room at constant temperature (21–24°C) and humidity (30–50%) on a 12/12 light/dark cycle, with white lights (light cycle) coming on at 2400 h and red lights (dark cycle) coming on at 1200 h. Mice were housed in the Department of Psychology at Columbia University. All procedures were conducted with approval from the Columbia University Institutional Animal Care and Use Committee (IACUC – Protocol No: AC-AAAG0054). At the end of the experiment, all animals were euthanized via decapitation with each individual’s brain and blood being stored for future analyses.

**Table 1:** Timeline of experimental procedures.

Day	Event
1-10	60 male CD-1 mice arrive and housed in standard sized cages in groups of 3 while habituating to facility
11-28	Mice remain housed in same groups of 3 and undergo standard behavioral testing
11	Open-field testing on the 30 subject mice, chosen randomly from the group of 60
14	Novel-object testing on the same 30 subject mice
19	All 60 mice habituated to the social test arena for 10 minutes
20	Social interaction testing; each subject mouse is paired with a novel stimulus mouse
25	Social approach-avoidance testing; each subject mouse is paired with a novel stimulus mouse (different partner to the previous social test)
29	30 subject mice placed in the vivarium and social group observations and census counts begin
29-48	Two hours of behavioral observations occur each day and census counts occur each day at threee separate time points: 2 h prior to dark cycle onset, 1 h post dark cycle onset, and 3 h post dark cycle onset
48	At the conclusion of the 2 h of behavioral observations, mice are euthanized via cervical dislocation and brains are collected for gene expression analysis

### Social Group Observations

Live behavioral observations were conducted in red light conditions for 2 h per day for 19 consecutive days by three trained observers, all observing at the same time in order to assure that all behaviors were accurately observed. Observations took place each day between 12 pm and 4 pm, during the first 4 h of the dark cycle. Behavioral observations were conducted as previously described (Williamson et al., 2016; **Table 2**), with additional recording of the location of each behavioral event (see Supplementary Figure S1). Observers were trained to recognize the unique ink patterns, and they are consistent with an exceptionally high degree of inter-rater reliability. 11 observers were used in total, each with a minimum of 50 h of coding experience prior to this study (mean 80 h).

**Table 2:** Ethogram of behaviors coded during vivaria observations.

Behavior	Description
Fighting	Individual lunges at or bites another individual
Chasing	Individual follows the target individual rapidly and aggressively whilst other individual attempts to flee.
Mounting	Individual mounts another individual from behind
Subordinate posture	Individual reacts to the movements of another individual by remaining motionless
Induced-Flee	Individual flees without any aggression shown by another individual

The total number of aggressive acts directed from one individual toward another were inputted into a frequency win/loss sociomatrix with winners in rows and losers in columns. As individuals cannot engage in agonistic interactions with themselves no data exists in the diagonal of each matrix. These data are referred to as directed or asymmetric data in social network analysis as individuals may direct behaviors more frequently to individuals than they receive from those individuals. From this, a binarized win/loss sociomatrix was calculated (see So et al., 2015; Williamson et al., 2016) for more information). Briefly, if individual *i* wins more contests against individual *j* than individual *j* wins against individual *i* then a 1 is allocated to the matrix cell [*i*,*j*] indicating that *i* dominates *j* and a 0 is allocated to the matrix cell [*j*,*i*] indicating that *j* is dominated by *i*. Following the rule proposed by Appleby, if there is a tie in the number of wins then both [*i*,*j*] and [*j*,*i*] are allocated a 0 (Appleby, 1983). Social network analysis was conducted using the binarized win/loss matrices. All statistical analyses were undertaken in R version 3.1.2 (R Core Team, 2015).

### Hierarchical Network Organization

Network metrics were calculated and analyzed using the ‘igraph v0.7.1,’ ‘sna v2.3-2,’ and ‘compete v0.1’ packages in R (Csardi and Nepusz, 2006; Butts, 2014; Curley, 2016a). The following network-level metrics were evaluated to assess hierarchical organization of the network: *(i) Density* – the proportion of all possible network ties that exist; *(ii) Average Path Length* – the mean number of steps between any two individuals in the network. Unreachable nodes are given the maximum path length; *(iii) Out-degree Centralization* – the degree to which the distribution of out-degrees across all individuals is skewed such that relatively few individuals have the highest out-degrees relative to the maximum possible. Individuals with high out-degrees dominate many other individuals. *(iv) Out-closeness Centralization* – the degree to which the distribution of out-closeness scores across all individuals is skewed such that relatively few individuals have the highest out-closeness relative to the maximum possible. Individuals with high out-closeness centrality are highly connected to many individuals in short steps (Freeman, 1978). *(v) Triangle transitivity* – this measure represents the proportion (Pt) of relations between all combinations of three individuals (A, B, C) in the network that are transitive (i.e., individual A dominates individual B, individual B dominates individual C, therefore individual A dominates individual C) (McDonald and Shizuka, 2013). This is scaled (t.tri) between 0 (the number of transitive three-way relations are no higher than random expectation) and 1 (all possible three-way relations are transitive as would occur in a completely linear dominance hierarchy). We tested for the significance of t.tri using a Monte-Carlo randomization of 1,000 generated random graphs using the method outlined by Shizuka and McDonald (2012) P-values are obtained by calculating the proportion of times that the randomly generated t.tri values are greater than the observed value. *(vi) Degree assortativity* **–** Out-degree and in-degree assortativity measure the extent to which individuals associate with other individuals that are of a similar out- and in- degree, respectively. Assortativity ranges between -1 (individuals of equivalent degrees never associate with each other) and 1 (individuals of equivalent degrees always associate with each other). We tested whether networks had significantly high assortativity by randomizing the degree distribution of each network 10,000 times. *P*-values are obtained by calculating the proportion of times that the randomly generated assortativity values are greater than the observed value (Newman, 2002, 2003; Noldus and Mieghem, 2015). *(vii) Maximum out-degree and minimum in-degree* – We also tested whether networks had a hierarchical structure by testing whether the maximum out-degree and minimum in-degree of each network significantly differed from that expected by chance. We computed the maximum out-degree and minimum in-degree for 5,000 random networks drawn from a Bernoulli graph distribution possessing the same number of individuals (nodes) and graph density as each network. Mean and standard deviations of *P*-values were obtained by comparing the proportion of times that the observed maximum out-degree and minimum in-degree were greater and lower, respectivelym than those values generated from the distribution of randomized networks for 20 replicates of each set of 5,000 randomizations (Butts, 2011). Networks were visualized using Gephi v0.8.2. Additionally, using the win-loss frequency sociomatrix, the following metrics of hierarchical organization were calculated and tested for their significance *(i) De Vries’ modified h’ value*, *(ii) steepness*, *(iii) directional consistency* (Williamson et al., 2016) using the ‘compete v0.1’ R package (Curley, 2016a).

### Network Communities

All analyses were undertaken using the ‘igraph v0.7.1’ R package (Csardi and Nepusz, 2006). To examine the community structure of the network, we first generated a symmetricized association matrix of all agonistic interactions by summing the frequency win/loss sociomatrix and its transpose. This represents the total number of interactions occurring between each pair of animals. The community membership of individuals is then determined using the Girvan-Newman method (Girvan and Newman, 2002; Newman and Girvan, 2004; Lusseau et al., 2008). Briefly, this method calculates the edge betweenness of all edges in the network and removes the edge with the highest value. Betweenness is recalculated for all remaining edges and the process continues until all edges are removed. The order in which edges are removed results in a hierarchically ordered dendogram. The modularity (*Q*) of each sub-division of each subgraph is calculated. *Q* is an index of how interconnected edges within each sub-division are compared to a random graph with *Q* = 0 representing that community ties are random. The sub-divisions that give the maximum value of *Q* for any graph represent the communities of the network. Following (Lusseau et al., 2008), to assess confidence in community membership assignment we bootstrapped our original data with replacement 1000 times. Each replicate had the same total number of observations as the original data. For each bootstrap replicate we reassigned community membership according to the Girvan-Newman community method. A community comembership matrix was then produced containing the total number of times that each pair of animals was assessed to be members of the same community out of the 1000 replicates. The community detection algorithm was then carried out on this comembership matrix to determine community membership. Differences in the frequency of aggressive behaviors between members of communities were assessed using Wilcoxon Signed Rank tests in R. We further tested community structure by applying non-metric multidimensional scaling (nMDS) to a distance matrix generated from a summary table of the total number of aggressive interactions in each vivarium by each individual.

### Non-agonistic Behavioral Observations

Census counts of the location of observable mice in the vivaria were undertaken daily at three time points (at 1000, 1300, and 1600 h). A trained observer recorded the identity of all visible mice in each vivarium at each time point. From these data we determined which individuals were in close association (within the same vivarium) at each census period. We then calculated a half-weight association index for each of the 435 dyads ranging between 0 and 1 (0 indicating that the animals were never associated and 1 that they were always associated) (Whitehead, 2008). Specifically, for two individuals *A* and *B*, their half-weight association index is calculated by HWI = x/(x + yAB + 0.5*(yA + yB)) where *x* = number of census periods where *A* and *B* are associated, *yA* = number of census periods with only *A* identified, *yB* = number of census periods with only *B* identified, *yAB* = number of census periods with *A* and *B* both identified but not associated. We also tested for a correlation between the association index matrix and the social network community comembership matrix using a Mantel Test using the vegan R package (Oksanen et al., 2015).

### Individual Network Position

All analyses were undertaken using the ‘igraph v0.7.1’ R package (Csardi and Nepusz, 2006). The following individual network measures were calculated: *(i) Out- and in-degree* – The number of ties directed to (out) and from (in) to each individual; *(ii) Out- and in-closeness* – A measure of how many individuals an individual directs connections to (out) or receives connections from (in) across relatively short paths; *(iii) Kleinberg’s Hub Score Centrality* – a measure of how influential an individual is to the network based upon the number of its outgoing ties (Kleinberg, 1999; So et al., 2015). Additionally, the rank order of individuals was assessed using the improved algorithm for the Inconsistencies and Strength of Inconsistencies (I&SI) ranking method (Schmid and de Vries, 2013; Williamson et al., 2016). Inter-correlations between network measures and ranks were analyzed using Spearman rank tests in R adjusting *p*-values for multiple comparisons using Holm’s method (Benton et al., 2013).

### Network Position and Pre-vivarium Behavior

Prior to housing in the vivarium, all 30 males underwent testing on two social and two non-social standard behavioral tests. The purpose of performing these tests was to determine whether measures of sociability and exploratory activity prior to being placed in a large social group corresponded in any way with dominance, network position, or community membership. All testing was conducted under red (dark phase) lighting conditions 1-6 h after lights off. The following tests were carried out: *(i) Open-Field***:** The open-field test is a behavioral assessment of exploratory activity in an unfamiliar environment (Prut and Belzung, 2003). Open-field testing was conducted as previously described (Champagne et al., 2009). *(ii) Novel Object:* The novel object test is typically described as a test of exploratory behavior (Crawley, 2007). Novel Object testing was conducted 3 days after the open-field test, in the same 59.5 cm × 59.5 cm square plastic box that subjects had previously been tested in. A novel object (small ceramic flower pot – height 3.8 cm, diameter 4.45 cm) was placed in the center of the open field. The subject mouse was removed from its home-cage and placed in the bottom-right corner of the box. The movement of the mouse through the arena as well as its interaction with the novel object was recorded with a video camera for 10 min. The mouse was then removed and returned to its home-cage. Fecal boli emitted during the test session were counted. The arena was cleaned with 70% ethanol between trials. Analysis of the video was completed using Observer (Noldus, V11.5). The frequency and durations of the following behaviors were coded: subject moving but not in proximity to the novel object, subject idle and not in proximity to the novel object, moving and in proximity to the novel object, idle and in proximity to the novel object, and sniffing the novel object. Proximity was defined as being within 7 cm of the novel object. *(iii) Social Interaction:* Social behavior was assessed using a social interaction test (File and Seth, 2003). This test was conducted 5 days after the novel object test. Day 1 consisted of a habituation phase. Mice were habituated to a square plastic box (31.75 cm × 27.3 cm) with pine bedding on the floor alone for 15 min. On Day 2, each mouse was placed in the corner of the box with another unfamiliar CD1 male mouse of the same age and approximate weight. The interactions between the mice were video recorded for 10 min. The mice were then removed and returned to their home-cages. Analysis of the video was conducted using Observer (Noldus, V11.5) with the time and duration of all behavioral events being coded (see Supplementary Table S1 for ethogram). *(iv) Social Approach-Avoidance:* Social behavior was assessed using the social approach-avoidance test (Crawley, 2007). This test was conducted 5 days after the social interaction test. Animals were first placed into a (31.75 cm × 27.3 cm) square plastic box for 10 min in order to habituate to the environment. The floor of the box was covered in pine bedding and contained two upside-down cups (height = 5.1 cm, diameter = 2.54 cm) placed in opposite corners. At the end of the 10 minute habituation phase, animals were removed from the box and returned to a holding cage. A novel object (plastic brick – height = 5.1 cm, width = 2.54 cm, length = 2.54 cm) was then placed under one cup and a neutral unfamiliar stimulus mouse (a male CD1 of the same age and approximate weight) was placed under the other cup. The mouse was then placed into the box for 10 min for the test phase and subsequently returned to his home cage. All testing was video recorded and conducted under red (dark phase) lighting conditions. Analysis of the video was conducted using Observer (Noldus, V11.5) with the time and duration of all behavioral events being coded (see Supplementary Table S2 for ethogram).

Following the guidelines for factor analysis in animal behavior research laid out by Budaev (Budaev, 2010), the Kaiser-Meyer-Olkin and Bartlett’s tests were used to determine sufficient sampling adequacy and parallel analysis was used to determine the appropriate number of factors for all analyses. Briefly, initial exploratory factor analyses were run for each behavioral test (open-field, novel-object, social interaction, social approach-avoidance) using the main behavioral variables coded in each test. Behavioral variables loading greater than 0.5 and less than -0.5 were considered to load onto each factor identified by parallel analysis (Supplementary Table S3). One behavioral variable from each factor from each test was then included in an overall factor analysis. As time spent sniffing all three body parts of the novel mouse loaded onto one factor in the social interaction test, we used total sniffing duration as a composite behavioral variable. The data included in the exploratory analysis for the social approach-avoidance test did not pass sampling adequacy so the most theoretically significant behavioral variable ‘Duration Sniffing Novel Animal’ was included in the overall factor analysis along with frequency of rearing. Variables with loading scores greater than 0.40 or less than -0.40 were considered as significant loadings in the overall factor analysis. We purposefully used selected variables from preliminary factor analyses to ensure the observation to variable ratio was kept to a minimum and was suitable for 30 subjects (Budaev, 2010). Factor scores were calculated using Thurstone’s method with the validity of score estimates being tested with the calculation of the maximum proportion of determinacy ρ2 (Grice, 2001). This is equivalent to the squared multiple correlation between each factor and original variables and should significantly exceed 0.5 for factor scores to be considered valid (Grice, 2001; Budaev, 2010). We then tested whether factor scores of behavior prior to being placed into the vivarium was associated with final network position or network position after day 4 using linear regression and Spearman Rank correlations. We also determined whether there was significant assortativity of individuals within the social network based upon factor scores. Finally, we assessed whether members of each community differed in their pre-vivarium behavior using Mann-Whitney tests. All factor analysis was carried out in R using the psych R package (Revelle, 2015).

### Network Position and Gene Expression

After the final behavioral observation, mice were immediately euthanized by cervical dislocation and brains removed and placed into hexane cooled by dry ice. Brains were stored at -80°C until dissection. Samples of the whole hippocampus (ventral and dorsal) and medial preoptic area (mPOA) were collected using a Harris Micro-Punch with reference to coronal cross-sections from the Mouse Brain Atlas (Paxinos and Franklin, 2004). The hippocampus was collected bilaterally from Bregma -0.82 mm to -1.46 mm and the mPOA was taken as one 1 mm diameter area along the midline from Bregma +0.14 mm to -0.7 mm. RNA was isolated from the hippocampus of each individual using the AllPrep RNA Micro Kit (Qiagen) and reverse transcribed to cDNA using the SuperScript III First-Strand Synthesis System for RT-PCR applications (Invitrogen). Quantitative RT-PCR was performed with 1 μL of cDNA using an ABI 7500 Fast Thermal Cycler and the Fast SYBR Green Master Mix reagent (Applied Biosystems). All primer probes (Sigma-Aldrich) were designed to span exon boundaries ensuring amplification of only mRNA. For each gene, *C*_T_ values were normalized to glyceraldehyde 3-phosphate dehydrogenase (GAPDH - endogenous control). Relative expression values were obtained by the ΔΔC_T_ method with fold-difference being determined respective to the average expression value for each gene in each brain region across all animals. The following validated quantitative PCR primers were used for mRNA analysis: GAPDH (Forward: TGTGTCCGTCGTGGATCTGA; Reverse: CCTGCTTCACCACCTTCTTGA), DNMT1 (For ward: GCCATGTGAACAGGAAGATGAC; Reverse: GTCCAAGTGAGTTTCCGGTCTT), DNMT3a (Forward: TCTTGAGTCTAACCCCGTGATG; Reverse: CCTCACTTTGCTGAACTTGGCT). Samples that did not yield sufficient RNA for cDNA conversion were eliminated from the analysis. Relative gene expression of each gene was compared to each measure of network position using Spearman rank correlations. To test for separate relationships between relative gene expression and dominance in each community, separate correlations were run if a linear model determined a significant interaction between community membership and network position. Outliers were determined using an iterated Grubbs Test (Grubbs, 1969), and results are reported for analyses including and excluding these outliers.

## Results

**FIGURE 1:**
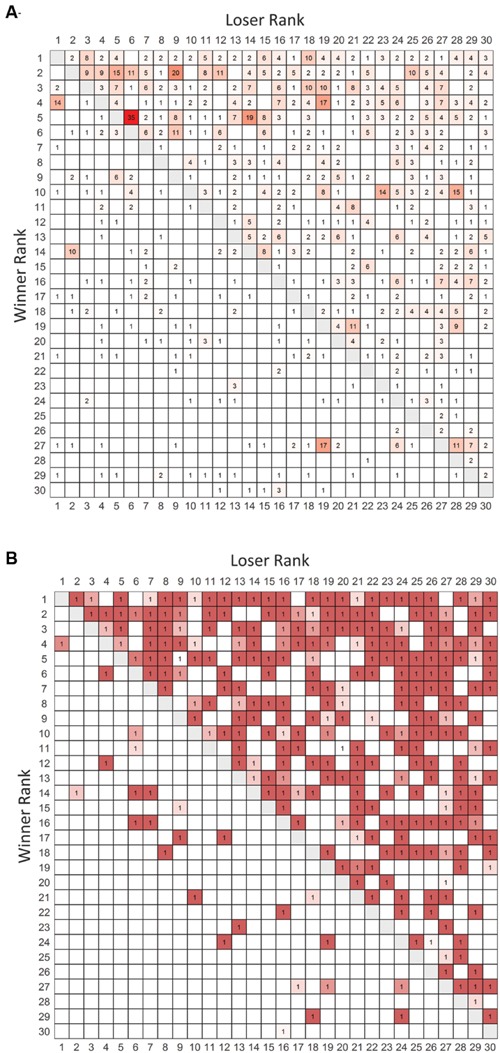
**Frequency and binarized win-loss sociomatrices. (A)** Total frequency of agonistic interactions between all pairs of individuals. Cells are colored from white (no wins) to red (highest number of wins). **(B)** Overall winners of each dyad are assigned a value of 1. Cells are colored from white to red with redness being directly related to the directional consistency of each dyad. Winners of each contest are listed in rows and losers are listed in columns. Rows and columns are in I&SI rank order.

We conducted observations for 38 h over 19 days which led to collection of data on 1230 agonistic interactions. The valued and binary sociomatrices for all aggression directed between pairs of animals living in the large vivaria are shown in **Figure 1**.

### Male Mice Establish a Hierarchically Organized Agonistic Social Network

The network of agonistic interactions has a low density (0.34), high average path length (2.12), high out-closeness centralization (0.54) and relatively high out-degree centralization (0.39) indicating that relationships are selective and that the power and influence within the network is unequally distributed. Congruently, randomization tests indicated the maximum out-degree was significantly higher than expected (*p* = 0.000 ± 0.000; mean ± SD from Monte-Carlo simulations) and the minimum in-degree was significantly smaller than expected (*p* = 0.003 ± 0.001) for random networks of the same size and density. Moreover, the out-degree assortativity (*r*_out_ = 0.28, *p* < 0.001) and in-degree assortativity (*r*_in_ = 0.26, *p* < 0.001) are both significantly positive indicating that individuals are more likely to connect to other individuals with a similar out-degree and in-degree meaning that the network has a core-periphery structure (Noldus and Mieghem, 2015). Triangle transitivity was also significantly higher than expected by chance (Pt = 0.94, t.tri = 0.76, *p* < 0.001) indicating a highly hierarchically organized network with minimal cyclic relationships. Hierarchical organization was confirmed by the significantly higher than chance values of Landau’s modified h’ (0.42, *p* < 0.001), directional consistency (0.79, *p* < 0.001) and steepness (0.31, *p* < 0.001)

### Male Mice Establish Distinct Social Network Communities

**FIGURE 2:**
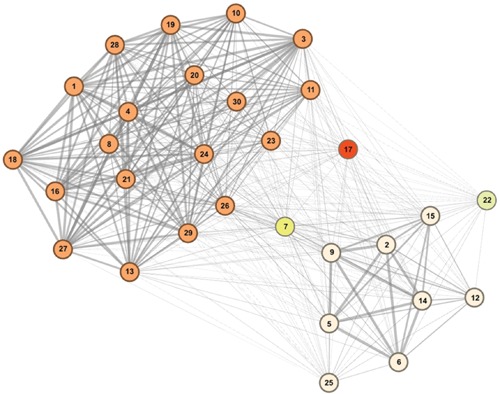
**Mice within overall social hierarchy establish separate hierarchically organized communities.** Community detection determined 19 individuals to belong to community A (orange), eight individuals to belong to community B (cream) and three individuals to not conclusively belong to either community. Tie strength is equivalent to the proportion of times that each subject pair were identified to belong to the same community from bootstrapped replications of original data (See Materials and Methods). Numbers refer to I&SI ranks.

**FIGURE 3:**
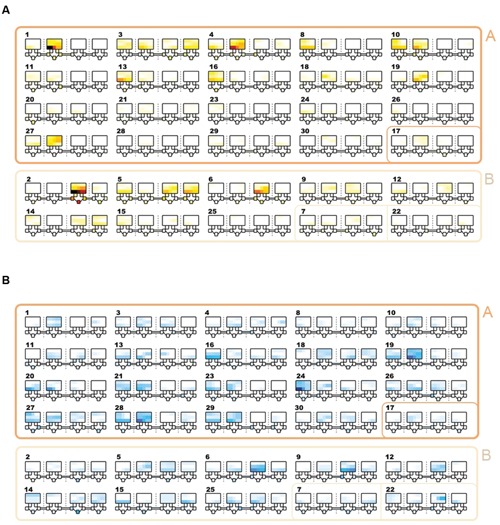
**Location and frequency of agonistic interactions by subject.** Schematics showing the frequency of aggressive contests that occurred in each vivarium. The largest squares refer to the top section of each of the four vivarium with each row representing the three shelves. Underneath each large square, five small squares represent the five nest-boxes in the bottom section of the vivarium. Tubes connecting vivaria 1-2, 2-3, and 3-4 are shown. Each number refers to the overall I&SI rank. IDs are ordered by community (A & A/other = rows 1-4; B & B/other = rows 5-6). Individuals in community A and B win and lose more frequently in vivaria 1&2 and vivaria 3&4, respectively. **(A)** Total frequency of wins. Colors range from white (0 fights won in location) through yellow and red to black. **(B)** Total frequency of losses. Colors range from white (0 fights lost in location) through light and dark blue.

**FIGURE 4:**
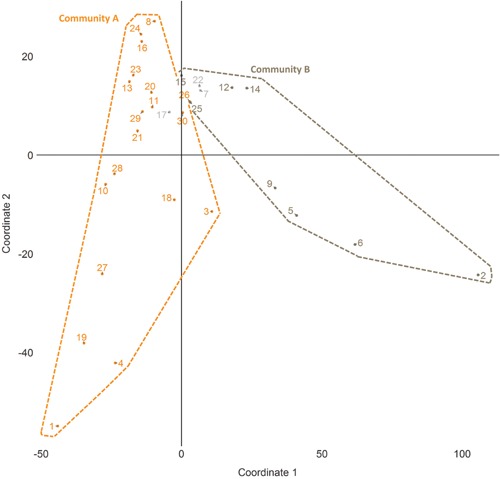
**Non-metric multidimensional scaling (nMDS) plot of individual space usage.** Scaling plot of the first two coordinates generated from nMDS analysis of the number of agonistic interactions undertaken by each subject in each vivarium. Numbers refer to I&SI ranks.

Community detection identified two major sub-communities within the overall agonistic network (Qmax = 0.24) (**Figure 2**). One consisted of 19 individuals (community A) and the other 8 individuals (community B). Additionally three individuals could not be placed within either community. Members of each community showed distinct preferences in the location of their agonistic interactions (**Figure 3**). Individuals from community A were more aggressive (Wilcoxon Signed-Rank Test: *V* = 180, *p* < 0.001) and received more aggression (*V* = 163, *p* < 0.001) in vivaria 1 and 2 compared to vivaria 3 and 4 (Supplementary Figure S2). Conversely, individuals from community B were more aggressive (*V* = 5, *p* = 0.078) and received more aggression (*V* = 0, *p* = 0.008) in vivaria 3 and 4 compared to vivaria 1 and 2. We confirmed this community structure by performing non-metric multidimensional scaling of total agonistic interactions of each individual by location (**Figure 4**). Notably, the most dominant individuals of each community are at the furthest extremes of each dimension with subordinate individuals from both communities more clustered close together. Further, the nMDS analysis indicated that two of the three extra individuals belonged to community B and one to community A (**Figure 4**).

### Network Community Structure Predicts Non-Aggressive Social Interactions

The half-weight association of each relationship was calculated from the census count data of non-agonistic social interactions that was obtained at three time-points each day. This measure gives an index of overall likelihood of social interaction of each pair of individuals. The average association index for dyads within communities (i.e., Community A – Community A dyads or Community B – Community B dyads) are significantly greater than for those between communities (i.e., Community A – Community B dyads; Wilcoxon Rank Sum Test: AA vs. AB – *W* = 20268, *p* < 0.001; BB vs. AB – *W* = 2705, *p* = 0.023, Supplementary Figure S3). Further, the difference between the medians of association indices occurring within and between communities are significantly larger than expected by chance as determined by 10,000 Monte-Carlo randomizations (*p* < 0.001). We also found that the half-weight association index matrix is significantly correlated with the community comembership matrix (Mantel Test – *r* = 0.38, *p* = 0.001). Therefore, community membership predicts social association between even non-agonistically interacting individuals.

### Male Mice Have Stable Individual Differences in Network Position and Power

**FIGURE 5:**
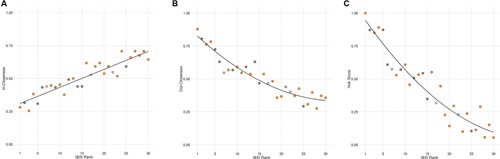
**Individual network positions area associated with dominance rank.** Individuals with a higher I&SI dominance rank have decreased in-closeness **(A)** and increased out-closeness **(B)** and hub scores **(C)** in the agonistic network. The best-fitting relationship is linear for (**A)** and quadratic for **(B)** and **(C)**. Each point represents one individual with color representing the network community of that individual (orange – community A, dark gray – community B, light gray – other).

The out-degree, in-degree, out-closeness, in-closeness, and hub score of each individual in the agonistic network were found to be highly significantly inter-correlated with each other as well as with the I&SI ranking of individuals (absolute rhos 0.78-0.99, all Holm’s *p* < 0.001). Dominant animals have higher out-degrees, out-closeness, and hub scores and lower in-degrees and in-closeness than subordinate animals (**Figure 5**). Notably, body weight prior to entering the vivarium, after removal or the change in body weight between these time points did not predict dominance rank or network position (all Holm’s adjusted *p* = 1).

Each individual’s Shannon’s evenness of the spatial distribution of giving or receiving aggression was not associated with network position or dominance rank (all *p* > 0.34). However, higher ranked individuals were significantly more likely to exhibit significant unevenness in their spatial distribution of giving aggression (Logistic Regression: β = -0.11 ± 0.06, *z* = -1.96, *p* = 0.049). Lower ranked individuals were significantly more likely to exhibit significant unevenness in the spatial distribution of receiving aggression (Logistic Regression: β = 0.12 ± 0.06, *z* = 2.04, *p* = 0.042). Across all individuals, there was no significant difference in evenness between giving and receiving aggression between days 1-6, but during days 7-12 (Wilcoxon Rank Sum test, *W* = 114.5, *p* < 0.001) and days 13-19 (*W* = 158.5, *p* < 0.001) giving aggression was significantly less equitable than receiving aggression (Supplementary Figure S4). This suggests that more dominant individuals become more localized in their space usage over time.

### Behavior Prior to Group Formation Predicts Early but Not Final Social Network Position

Factor analysis of the behavior exhibited by each mouse on the four standard tests conducted prior to vivarium housing resulted in two main factors which we named ‘activity’ and ‘exploration’ (see Materials and Methods). Activity and exploration accounted for 25 and 21% of total variance in behavior, respectively. Network position (out-degree, in-degree, in-closemess, out-closeness, or hub score) was not related to each individual’s activity or exploration factor scores (all *R*^2^< 0.035). Further, individual activity (*r* = -0.04, *p* = 0.67) or exploration (*r* = 0.00, *p* = 0.19) scores did not predict assortativity in the overall network, though at the community level, we did find that eventual members of community A were significantly less active in pre-vivarium behavioral tests than eventual members of community B (Mann-Whitney Test, *W* = 25, *p* = 0.005, Community A median = -0.35 (IQRs: -0.67–0.36), Community B median = 0.85 (IQRs: 0.28–1.40).

As behavior pre-vivarium may be more reflective of initial behavior in the vivarium we examined if individual network position at the end of Day 4 was associated with pre-vivarium behaviors. Early out-degree (ρ = -0.41, *p* = 0.025) and out-closeness (ρ = -0.44, *p* = 0.016) were significantly negatively associated with exploration scores whereas in-degree (ρ = 0.39, *p* = 0.034) and in-closeness (ρ = 0.38, *p* = 0.039) were significantly positively associated with exploration scores. No relationship between early network scores and activity scores were found. Notably, time spent sniffing the novel animal in both the social interaction and social approach tests was negatively associated with early out-degree (SI: ρ = -0.48, *p* = 0.007; SA: ρ = -0.54, *p* = 0.002) and out-closeness (SI: ρ = -0.47, *p* = 0.008; SA: ρ = -0.55, *p* = 0.001), but time spent sniffing the novel object or time in the inner area of the open-field was not (Supplementary Figure S5).

### Hippocampal Expression of Plasticity Related Genes Are Associated With Network Position

**FIGURE 6:**
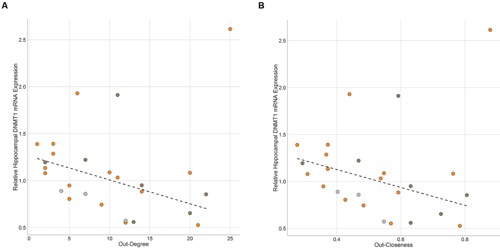
**Brain Gene Expression and Social Dominance.** Hippocampal DNMT1 expression is negatively associated with **(A)** Out-Degree and **(B)** Out-Closeness. Black hashed lines represent best-fit with outlier removed. Each point represents one individual with color representing the network community of that individual (orange – community A, dark gray – community B, light gray – other).

DNMT1 gene expression in the hippocampus was significantly negatively associated with out-degree (ρ = -0.40, *p* = 0.042) and hub score (ρ = -0.40, *p* = 0.042) and marginally negatively associated with out-closeness (ρ = -0.35, *p* = 0.080; **Figure 6**). One individual with the highest DNMT1 gene expression value was determined to be an outlier using an iterated Grubbs Test. This individual had the highest out-closeness score. He was an alpha male that rarely lost any fights until the last 3-4 days of observations when he began to lose a series of fights to one other dominant individual. Removing this outlying data point leads to much higher significant negative associations between DNMT1 gene expression and out-degree (ρ = -0.58, *p* = 0.003), out-closeness (ρ = -0.52, *p* = 0.008) and hub score (ρ = -0.58, *p* = 0.003). In-degree and In-closeness were not related to hippocampal DNMT1 expression, though there was a trend for a positive association with the outlier removed (in-degree: ρ = 0.36, *p* = 0.078; in-closeness: ρ = 0.37, *p* = 0.072). Across all individuals in the network, hippocampal DNMT3a expression was not associated with any network measure. However, when examining each community (as defined in **Figure 4**) separately, out-degree (ρ = -0.71, *p* = 0.057), out-closeness (ρ = -0.73, *p* = 0.040) and hub score (ρ = -0.74, *p* = 0.046) were negatively associated with DNMT3a expression amongst community B individuals (Supplementary Figure S6). No relationship was observed among community A individuals. There was no significant relationship between DNMT1 or DNMT3a and network measures in the medial preoptic area (Supplementary Figure S7).

## Discussion

### Mice Establish a Hierarchically Organized Dominance Network

We found that a group of 30 communally living male outbred CD1 mice formed a remarkably hierarchically organized social dominance network. The agonistic social network had a very low overall density, high average path length, and high out-closeness centralization. These features demonstrate that the power within the network is disproportionately distributed with most network power being monopolized by relatively few individuals. The triangle transitivity was also significantly higher than chance evidencing a highly linear hierarchical structure (Shizuka and McDonald, 2015). Degree assortativity and out-degree assortativity were also significantly positive indicating that individuals were more likely to be connected to other individuals of similar out- and in-degrees, indicating that there exists a core-periphery structure to the social network (Noldus and Mieghem, 2015). These findings were consistent with the highly significant Landau’s modified h’, steepness and directional consistency values that indicated that the social hierarchy was both highly linear and steep. These results extend our previous findings that male CD1 mice living in groups of 12 form hierarchically organized dominance networks (So et al., 2015; Williamson et al., 2016). The observed degree of linearity are also similar to those observed in other non-primate mammalian societies with equivalent group sizes (Chase, 1980; Fournier and Festa-Bianchet, 1995; Chase and Seitz, 2011; Sigurjonsdottir et al., 2012).

We also found that mice further organized themselves into network communities using the Newman-Girvan modularity matrix clustering algorithm. This approach has been well developed and validated for identifying community structure in species as diverse as whales, dolphins, birds and primates (Girvan and Newman, 2002; Lusseau and Newman, 2004; Lusseau et al., 2008; Griffin and Nunn, 2011; Aplin et al., 2013). Based upon the frequency of agonistic interactions, we found strong evidence for two main communities comprised of 19 and 8 individuals, respectively. It was not possible to identify with certainty using this method the community membership of the other three remaining individuals. Individuals in the larger community A were more likely to engage in aggressive interactions with each other and focused these interactions within vivaria 1 and 2. Individuals in the smaller community B were also more likely to be aggressive towards one another with these interactions more commonly occurring in vivaria 3 and 4.

These results were further confirmed and extended by applying a nonmetric multidimensional scaling to the frequency of agonistic interactions in each vivaria by each individual. This strongly agreed with the finding that there were indeed two main communities of mice that could be identified based upon space usage. This analysis was also able to identify the community membership of the remaining three individuals. These community memberships were also confirmed by non-agonistic data. More dominant individuals were at the extremes of the nMDS plot and more subordinate individuals were closer to the boundary of the two clusters. Dominant individuals were also more likely to show significant unevenness in their utilization of those locations where they attack other individuals. This unevenness also increased over time. This is highly suggestive that more dominant individuals were attempting to form territories, a finding consistent with previous reports that male wild mice living in large semi-natural environments will form territories which they will seek to defend from intruders (Mackintosh, 1970; Crowcroft, 1973; Hurst et al., 2001; Perony et al., 2012).

Using our daily census counts of mice, we found that the average half-weight association index for those relationships within each network community was significantly higher than for between community relationships. Community membership and association index matrices were also significantly correlated with one another demonstrating that these network communities are not only related to the aggressive interactions between mice but to their overall social lives.

We demonstrate in this study that by providing sufficient space that it is possible to collect social behavior data on a large group of laboratory mice that can then be used to determine and assess changes in the social network patterning at the individual, relationship and group structural level in the laboratory. Using such data we are able to show that mice navigate social environments that vary over time and are spatially complex. Understanding how mice manage and maintain their multiple social relationships across time and social contexts enables us to gain insight into the neurobiological processes underlying social learning and competence that are integral aspects of healthy social functioning for all species (Taborsky and Oliveira, 2012; Hofmann et al., 2014; Cardoso et al., 2015; Fernald, 2015).

### Behavior Prior to Group Formation Does Not Predict Individual Network Position

In the directed agonistic network, individuals with high out-degree, out-closeness and hub-score were indicative of more powerful and socially dominant individuals. Individuals with higher in-degree and in-closeness scores were more subordinate. These network metrics were highly inter-correlated with each other likely due to the highly organized network structure. We confirmed the accuracy of these metrics for assessing social power by demonstrating that they correlated extremely highly with the dominance ranking produced using the I&SI ranking algorithm (Schmid and de Vries, 2013).

Assessing the behavior of mice on standard laboratory tests of social and non-social behavior prior to group housing, we found two factors which we named “activity” and “exploration” that significantly accounted for a large proportion of the variance in behavior. The two factors were not related to one another congruent with other studies (Berton et al., 1997). Other research in laboratory mice supports our finding that motor activity levels of individual mice are consistent across time and in different contexts indicative of a robust personality trait (Paulus et al., 1999; Tang et al., 2002). The behavioral variable that most strongly correlated with “exploration” factor scores was time spent sniffing in the social interaction test though all other variables also correlated with “exploration” factor scores more than *r* = 0.4 (Supplementary Table S4). We therefore did not clearly observe a distinction between social and asocial exploration as others have noted (Makino et al., 1991; Berton et al., 1997; Maier et al., 1988), although the highest correlation observed between exploration behavior variables was between time spent sniffing the novel animal in the two social tests. Others have reported similar associations between these two tests in mice (Brodkin, 2007; Crawley, 2007).

Previous studies have suggested both positive (Boogert et al., 2006; David et al., 2011) and negative associations (Verbeek et al., 1999; Fox et al., 2009) between activity levels/exploration and dominance rank. It has been argued that ecological, social and life-history contextual factors may mediate the relationship between these variables (Dingemanse and de Goede, 2004). In this study, we could not find any relationship between any behavioral measure made prior to group-formation and eventual social network position. Our data are consistent with one other study that found no pre-group formation differences in activity, anxiety-like or exploratory behavior between male mice that would later become dominant and subordinate in groups of five (Hilakivi-Clarke and Lister, 1992). We did, however, find that investigation of novel social stimuli prior to group formation was negatively associated with initial out-degree and out-closeness in the social network. This provides evidence that these standard tests of social behavior do reliably measure a social phenotype that is related to initial social approach behavior and might suggest that social behavior styles prior to group formation can modulate early social interactions in groups. However, these tests are not reliable for predicting long-term social behavior of animals in social networks being not related to ultimate social network position or dominance rank suggesting that as the group context changes these initial behavior styles become less important than concurrent experiential factors for governing social interaction (Hsu et al., 2006; Chase and Seitz, 2011).

We also found that animals of similar activity or exploration factor scores did not preferentially assort or disassort with one another in the social dominance network. Homophily, the preferential association of phenotypically similar individuals, has been observed in human and animal social groups (McPherson et al., 2001). For instance, Aplin et al. (2014) found in a natural population of great tits that they assort their social interactions based on their exploratory personality type. Chimpanzees and baboons also assort based on personality measures such as sociability and boldness (Massen and Koski, 2014; Carter et al., 2015). In our social system, however, it seems most likely that the social dominance structure of the population is most critical to determining the associations of individuals as we did find that animals showed significant in-degree and out-degree assortativity. Curiously, we found that the pre-group-formation activity levels of individuals were significantly lower in individuals in community A compared to community B. It is not immediately clear why individuals of lower activity levels would exist in larger communities and more activity in smaller communities, though it has been argued that the number and strength of ties in a social network may relate to personality type (Pike et al., 2008; Croft et al., 2009). It is possible that the less active mice remained in the main large community and more active mice split from it, but this hypothesis requires further investigation.

Our findings have significant implications for social behavior research carried out in laboratory mice. Over the last decade, the majority of work on social behavior of mice has utilized short and simple behavioral battery tests, often using only one outcome behavioral parameter (Peters et al., 2015). This behavioral assay approach fails to incorporate the complexity of any behavior but especially social behavior. The social approach-avoidance test which exists in several different guises (Yang et al., 2001; Moy et al., 2004; Nadler et al., 2004) and the social interaction test (File and Seth, 2003) are the most commonly used social behavior assays in laboratory mice. Both use the total time spent sniffing the novel animal as an index of the sociality of the subject animal. We would argue, as others have (Hofmann et al., 2014; Peters et al., 2015), that it is not clear whether exhibiting high or low social investigation in these tests is a reliable indicator of something as complex as social behavior. It is possible that the investigation of novel individuals in a novel environmental context is actually more related to behavioral inhibition or exploratory behavior than social behavior. Indeed, our findings that time spent investigating both social and non-social stimuli are grouped together in the same ‘exploration’ factor would seem to support the hypothesis that these tests are not specific to social behavior. It is also not clear from our results that these social behavior assays have strong predictive value for the social behavior of individuals in a group context. Therefore, we suggest that there is a much larger and more complex aspect of the social lives of mice that is not captured by these tests and ought to be considered when investigating the effects of genetic or pharmacological treatments on social behavior.

### Social Network Position is Associated With Differential Brain Gene Expression

Hippocampal DNMT1 mRNA expression levels are significantly negatively related to network measures of power and dominance (i.e., out-degree, out-closeness, hub score) across all individuals. The hippocampus is critical for the integration of social information and regulation of learning about social status (Curley et al., 2011; van der Kooij and Sandi, 2012). While DNMT1 has traditionally been viewed as important for the maintenance of DNA methylation, it is expressed at high levels in the adult hippocampus (Brown et al., 2008), and recent studies have found that expression of DNMT1 dynamically shifts in relation to differential environmental experiences that may be related to aggression (Zhang et al., 2010; Gudsnuk and Champagne, 2012; Kundakovic et al., 2013). Additionally, studies have shown that variation in social experience can be associated with changes in DNA methylation patterns that are dependent upon the activity of DNA methyltransferases (Elliott et al., 2010; Borghol et al., 2012; Provençal et al., 2013; Alvarado et al., 2014; Alvarado et al., 2015). Specifically, chronic social defeat stress leads to long-term demethylation of the *Crf* promoter in mice and consequently leads to an increase in social avoidance behaviors (Elliott et al., 2010) In cichlid fish, social crowding during development results in decreased methylation of the GnRH1 gene (Alvarado et al., 2015) and pharmacological induction of increased methylation leads to development of socially dominant individuals while pharmacological inhibition of DNMT activity leads to development of socially subordinate individuals (Lenkov et al., 2015). Taken together with our findings it is plausible that changes in social network position and social status may be regulated via DNA methyltransferase-dependent epigenetic mechanisms in the hippocampus.

Higher levels of DNMT1 in more subordinate less powerful mice may suggest that these mice are experiencing a social suppression of gene expression in the hippocampus. Subsequent differences in gene expression between more and less dominant individuals in a brain region specific manner may enable individuals of different social statuses to learn how to express socially contextually appropriate behaviors (Cardoso et al., 2015). Interestingly, the individual with the highest DNMT1 mRNA expression was an extremely dominant individual who lost a number of fights to one other dominant male immediately prior to the end of observations requiring them to learn to express subordinate behavior in a socially specific manner.

While there was no overall relationship between DNMT3a expression and dominance and social network measures, it is relevant to note that we found that more subordinate individuals in the smaller community B exhibited greater levels of relative DNMT3a mRNA expression than dominant individuals. DNMT3a is well known to functionally modulate the effect of environmental experiences on brain gene expression and specifically regulate learning about socioemotional behavior including social defeat (Yu et al., 2011; Hammels et al., 2015). In honeybees, inhibition of DNMT3a in larvae leads to their development as a queen bee (Evans and Wheeler, 1999; Kucharski et al., 2008), further demonstrating that DNMT3a can play a plastic role in regulating social roles. Although we do not see this association in the larger community, this may be due to temporal differences in when each community is undergoing changes in social roles and thus brain plasticity, or related to differential social dynamics (e.g., the higher rate of repeated social interactions) that occur in small versus large communities.

Finally, although changes in the DNA methylation of specific genes (e.g., *GnRH1*) in the mPOA is integral for the ability to transition from dominant to subordinate status in cichlid fish undergoing social ascent (Maruska and Fernald, 2011), we found no relationship between social network position or rank and DNMT1 or DNMT3a mRNA expression in the mouse mPOA, suggesting that plasticity in the mPOA may not be important to the maintenance of social status in mouse stable hierarchies.

We have demonstrated that a large group of 30 male mice form a hierarchically organized agonistic social network. This network is further sub-organized into two main network communities that are spatially dissociated. We also demonstrate that behavior of males prior to group-formation in commonly used laboratory behavior tests (the open-field test, novel object test, social interaction test, and approach-avoidance paradigm), is not predictive of later social network position though is somewhat associated with initial behavior in the network prior to the group stabilizing its hierarchical organization. We further show that dominance rank and network position are associated with differential hippocampal DNMT1 and DNMT3a expression suggesting that increased hippocampal neural plasticity may be associated with the development of contextually specific subordinate behavior. Future studies will need to mechanistically address the functional significance of changes in hippocampal DNMT expression in regulating social competence within a social hierarchy. Studying the neurobiology of complex social behavior of mice requires the development of improved paradigms of behavioral assessments that go beyond mice interacting in dyads in novel contexts for brief periods of time. Here, we have shown that using ethologically relevant housing of male mice over 3 weeks is sufficient to reveal complex spatiotemporal patterns of agonistic behavior between male mice with context-specific consequences for brain gene expression.

## Author Contributions

CW and JC designed the experiment and wrote the paper. BF, JC, and CW carried out statistical analyses. CW collected data.

## Conflict of Interest Statement

The authors declare that the research was conducted in the absence of any commercial or financial relationships that could be construed as a potential conflict of interest.
