# Supplementary material for: Mouse Social Network Dynamics and Community Structure are Associated with Plasticity-Related Brain Gene Expression
Source: Front Behav Neurosci. 2016 Aug 4;10:152. doi: 10.3389/fnbeh.2016.00152 (PMC4972826; doi:10.3389/fnbeh.2016.00152)

**SUPPLEMENTARY MATERIAL**

**Supplemental Table 1. Ethogram of Behaviors Coded in the Social Interaction Test**

| **Behavior** | **Description** |
| --- | --- |
| Idle/nothing | Individual is not moving or interacting with other individual |
| Sniff head | Individual approaches and makes an olfactory investigation of the head of other individual |
| Sniff body | Individual approaches and makes an olfactory investigation of the body of other individual |
| Sniff anogenital | Individual approaches and makes an olfactory investigation of the anogenital region of other individual |
| Sniff and follow | Individual approaches and makes an olfactory investigation of other individual while following around the space |
| Rearing | Individual rears on hind legs |
| Digging | Individual digs into pine bedding on floor |
| Self-grooming | Individual grooms self with mouth and/or paws |
| Jumping | Individual jumps |
| Contact side by side | Individual has contact with other individual while neither sniffing nor biting |
| Pursuing | The focal individual follows the target individual rapidly and aggressively whilst the target individual attempts to flee |
| Allogrooming | Individual grooms with their paws and mouth the fur and/or face of another individual |
| Biting | Individual bites other individual |
| Lunging | Individual moves towards other individual as if to attack |
| Tail Rattle | Individual displays a fast tail vibration of the tail, often observed in a distance ambivalence situation |
| Defensive freeze | Individual freezes as other individual moves to attack |
| Display of subordinate posture | Individual reacts to the movements of the partner by remaining motionless |
| Fleeing | Individual moves rapidly away from the partner |

**Supplemental Table 2. Ethogram of Behaviors Coded in the Social Approach-Avoidance Test**

| **Behavior** | **Description** |
| --- | --- |
| Near Novel Object | Individual is close to cup containing the novel object but not sniffing or engaging with it |
| Sniff Novel Object | Individual is sniffing the cup containing the novel object |
| Near Social Stimulus | Individual is close to the cup containing the social stimulus but not sniffing or engaging with it |
| Sniff Social Stimulus | Individual is sniffing the cup containing the social stimulus |
| Rearing | Individual rears on hind legs |
| Idle/Nothing | Individual is not moving or interacting with other individual or the novel object |
| Moving | Animal is moving through the space but not engaging with social stimulus or novel object |
| Digging | Individual digs into pine bedding on floor |
| Self-grooming | Individual grooms self with mouth and/or paws |
| Jumping | Individual jumps |

**Supplemental Table 3. Summary of Factor Loadings for Each Standard Behavioral Test**

**a)** Open-Field, KMO = 0.53, Bartlett’s Test p<.001

| **Behavior** | **Factor 1** | **Factor 2** |
| --- | --- | --- |
| Latency to Center of Area (s) | 0.03 | **0.97** |
| Rearing Frequency | **-0.59** | -0.48 |
| Duration Immobile (s) | **0.99** | 0.11 |
| Duration in Inner Area (s) | -0.01 | **-0.62** |
| Number of boli | -0.20 | 0.03 |
| Proportion Variance | 0.27 | 0.31 |

**b)** Novel-object, KMO = 0.55, Bartlett’s Test p<.001

| **Behavior** | **Factor 1** | **Factor 2** |
| --- | --- | --- |
| Latency to Move Near (s) | **0.99** | -0.14 |
| Latency to Sniff Near (s) | **0.64** | -0.08 |
| Duration Move Near (s) | -0.10 | **0.71** |
| Duration Sniff Near (s) | 0.02 | **0.73** |
| Time immobile (s) | 0.36 | **-0.54** |
| Number of boli | -0.10 | 0.43 |
| Proportion Variance | 0.26 | 0.26 |

**c)** Social Interaction, KMO = 0.61, Bartlett’s Test p<.001

| **Behavior** | **Factor 1** | **Factor 2** |
| --- | --- | --- |
| Duration Sniff Anogenital (s) | 0.20 | **0.83** |
| Duration Sniff Body (s) | 0.09 | **0.71** |
| Duration Sniff Follow (s) | -0.08 | 0.19 |
| Duration Sniff Head (s) | 0.27 | **0.50** |
| Time immobile (s) | **0.88** | 0.07 |
| Frequency of Rearing | **-0.92** | -0.11 |
| Proportion Variance | 0.29 | 0.25 |

**Supplemental Table 4. Correlation of Behavioral Variables and Factor Scores**

OF: open-field, NO: novel object, SI: social interaction, SA: social approach/avoidance

| **Behavior** | OF: Duration in Inner Area (s) | NO: Duration Sniffing Novel Object (s) | SI: Duration Sniffing Novel Animal (s) | SA: Duration Sniffing Novel Animal (s) |
| --- | --- | --- | --- | --- |
| Exploration Factor Score | **0.55**** | **0.48**** | **0.94***** | **0.43*** |
| OF: Duration in Inner Area (s) |  | 0.19 | 0.33ⱡ | 0.22 |
| NO: Duration Sniffing Novel Object (s) |  |  | **0.37*** | 0.11 |
| SI: Duration Sniffing Novel Animal (s) |  |  |  | **0.44*** |

**a)** Exploration - *** p<.001, **p<.01, *p<.05, ⱡp<.10

**b)** Activity - *** p<.001, **p<.01, *p<.05

| **Behavior** | OF: Rearing Frequency | SI: Rearing Frequency | SA: Rearing Frequency |
| --- | --- | --- | --- |
| Activity Factor Score | **0.85***** | **0.73***** | **0.73***** |
| OF: Rearing Frequency |  | **0.50**** | **0.45*** |
| SI: Rearing Frequency |  |  | **0.41*** |

**Supplemental Figure 1. Housing Vivaria** - View of all 4 inter-connected vivaria (V1, V2, V3, V4) connected via a long tube (Tube B) across the room (Left). View of connection between the nest-boxes of adjacent vivaria V1 & V2 connected via a short tube (Tube A) (Bottom Right). An identical tube (Tube C) connects V3 & V4. View of vivaria V3 and V4 showing food and water at top of upper section and nest-boxes underneath.

**
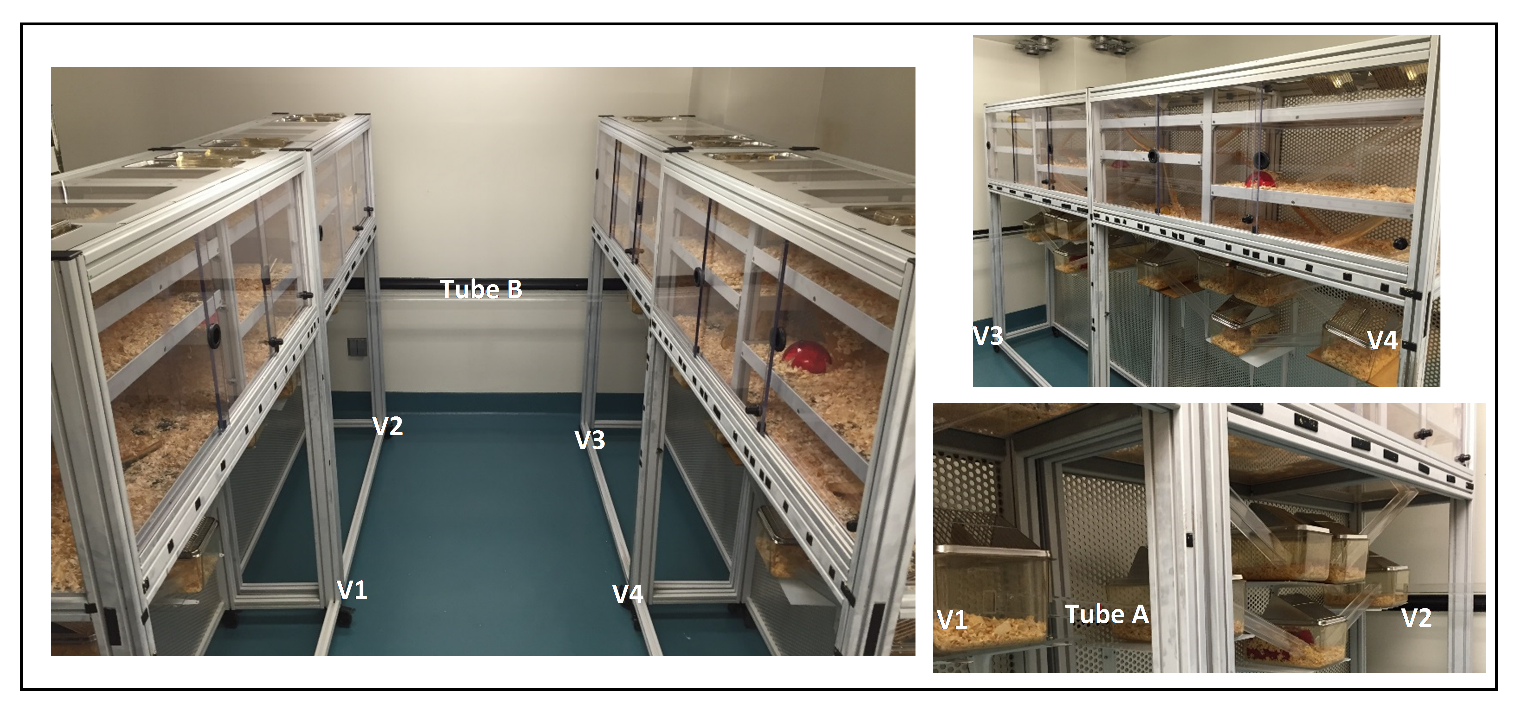
**

**Supplemental Figure 2. Location of Agonistic Interactions By Community** Boxplots of the total number of aggressive interactions that occurred in vivaria 1-2 or 3-4 separated by community (A, B or Other). Each point refers to a unique individual.

**
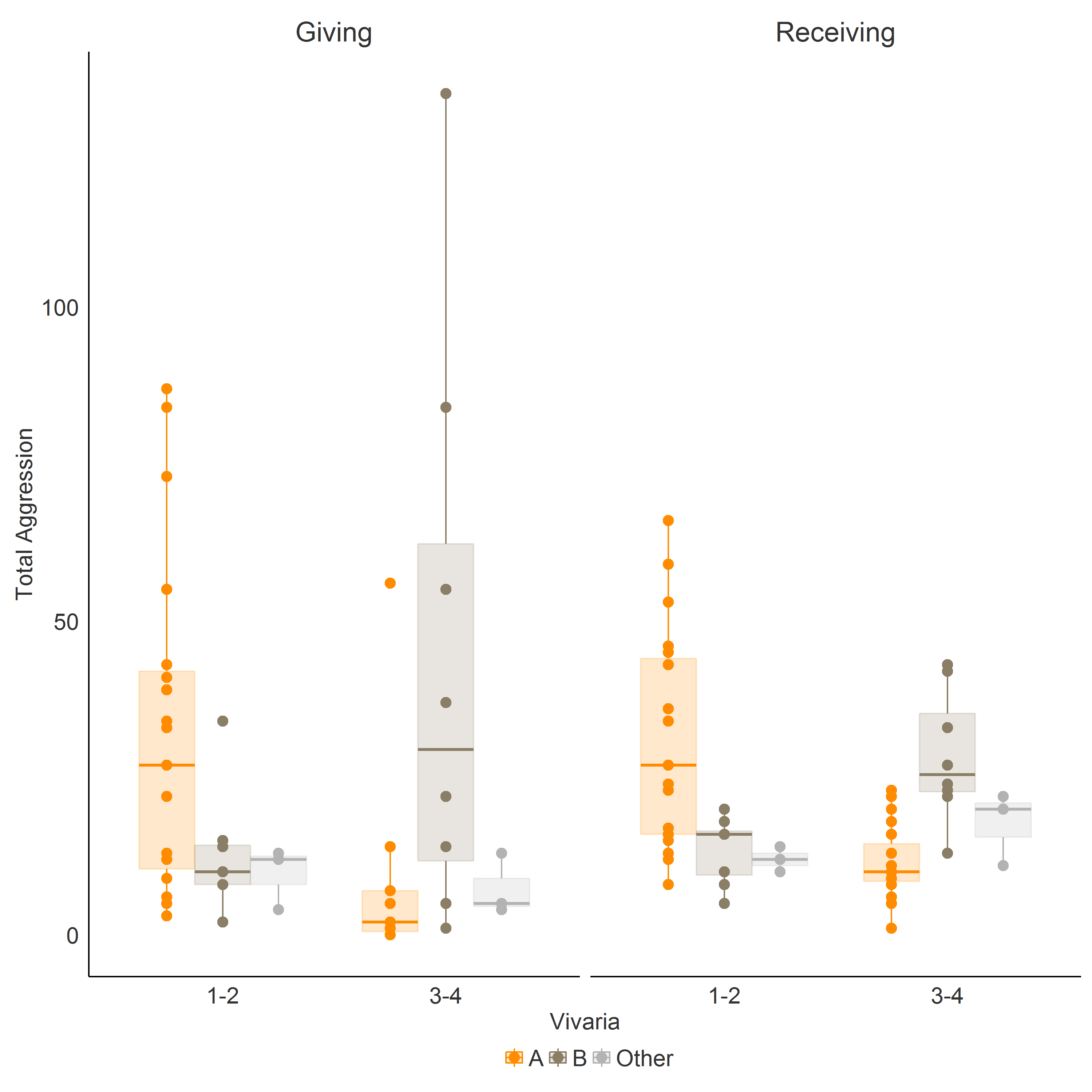
**

**Supplemental Figure 3. Half-Weight Association Indices Within and Between Communities** Boxplots of the half-weight-association indices (HWI) occurring between individuals in community A (AA) or community B (BB) or between individuals in each community (AB).

**
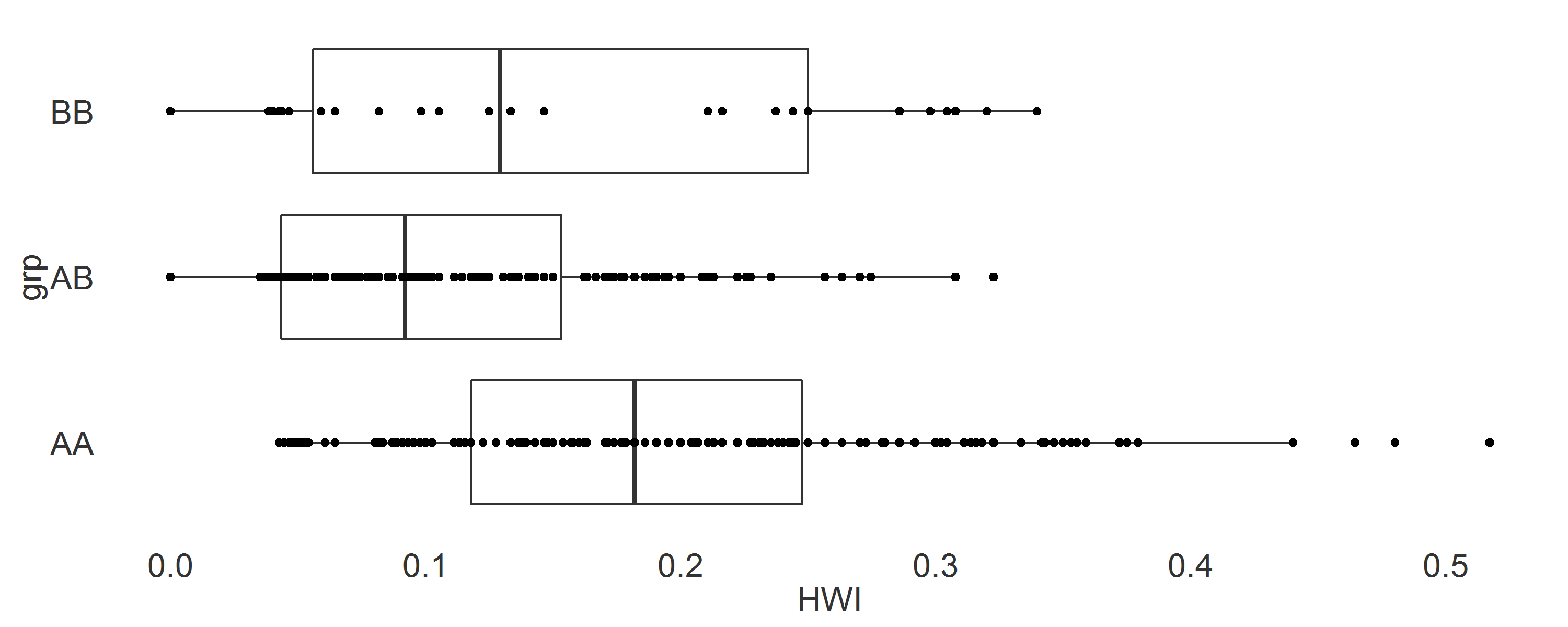
**

**Supplemental Figure 4. Changes in Space Usage Evenness of Giving and Receiving Aggression by Days** Boxplots of Shannon’s Evenness of giving and receiving aggression of individuals over days.


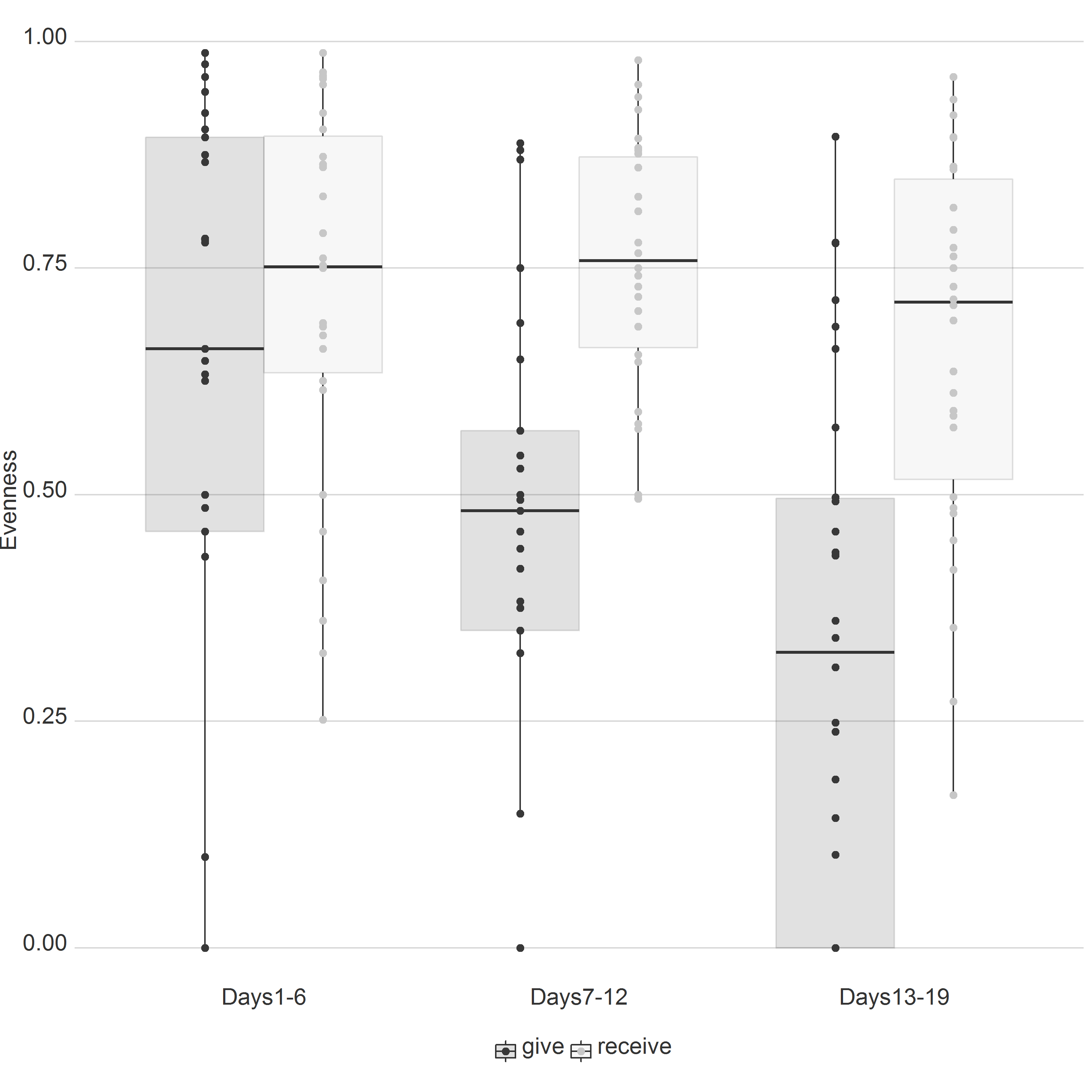


**Supplemental Figure 5. Investigation of novel social stimuli are negatively associated with Initial Individual Network Position**. Individuals who investigate a novel social animal for longer on (A) the social interaction test and (B) the social approach test have a smaller out-degree after four days of group formation. No relationship between out-degree and behavior on (C) the novel object or (D) the open-field tests were found. Behavior on all tests was not related to final network position. Each point represents one individual with color representing the network community of that individual (orange – community A, dark gray – community B, light gray – other).


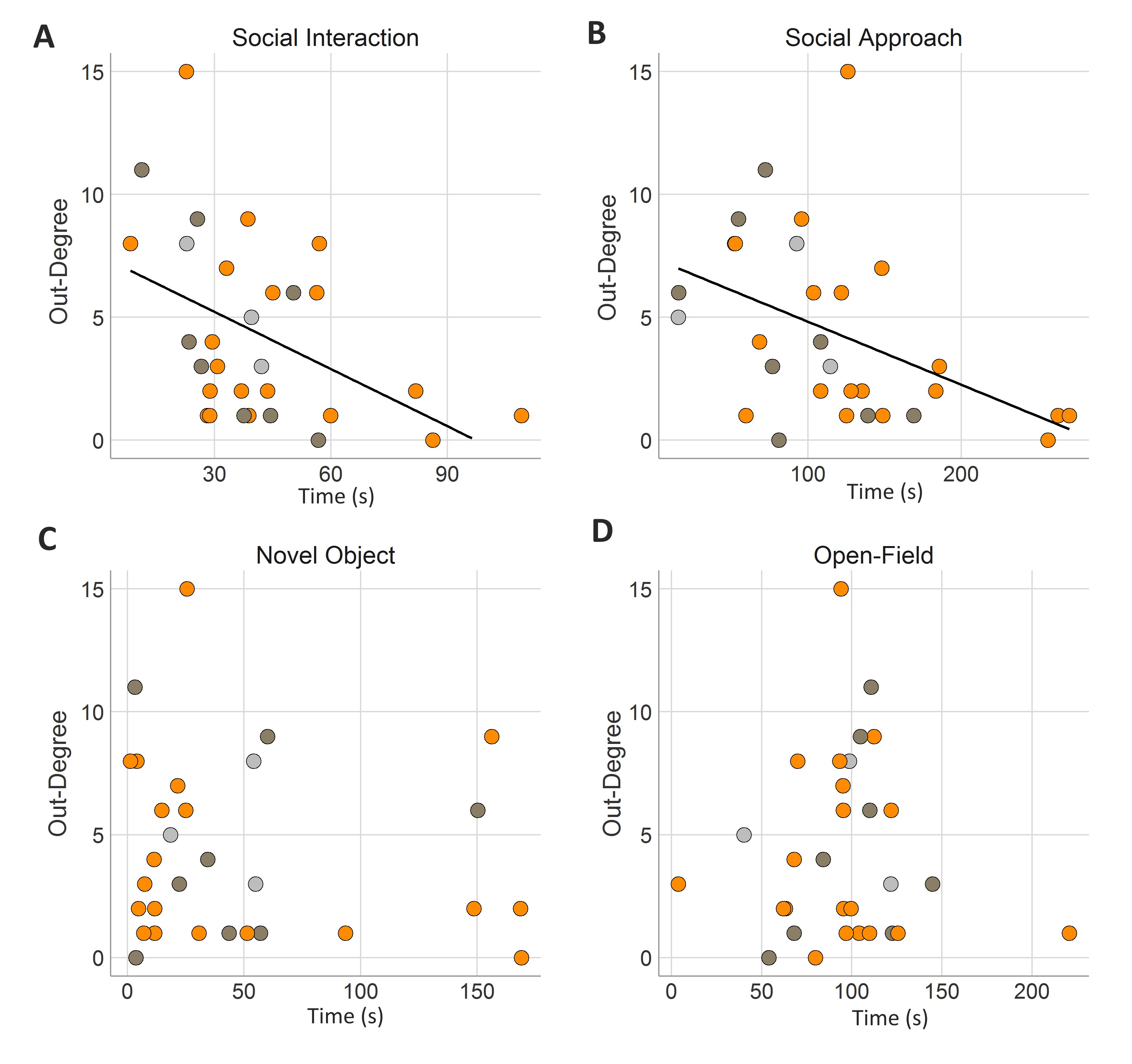


**Supplemental Figure 6.** Hippocampal DNMT3a expression is negatively associated with Out-Closeness in individuals in Community B but not Community A. Trendline represents the line of best with the outlier removed.


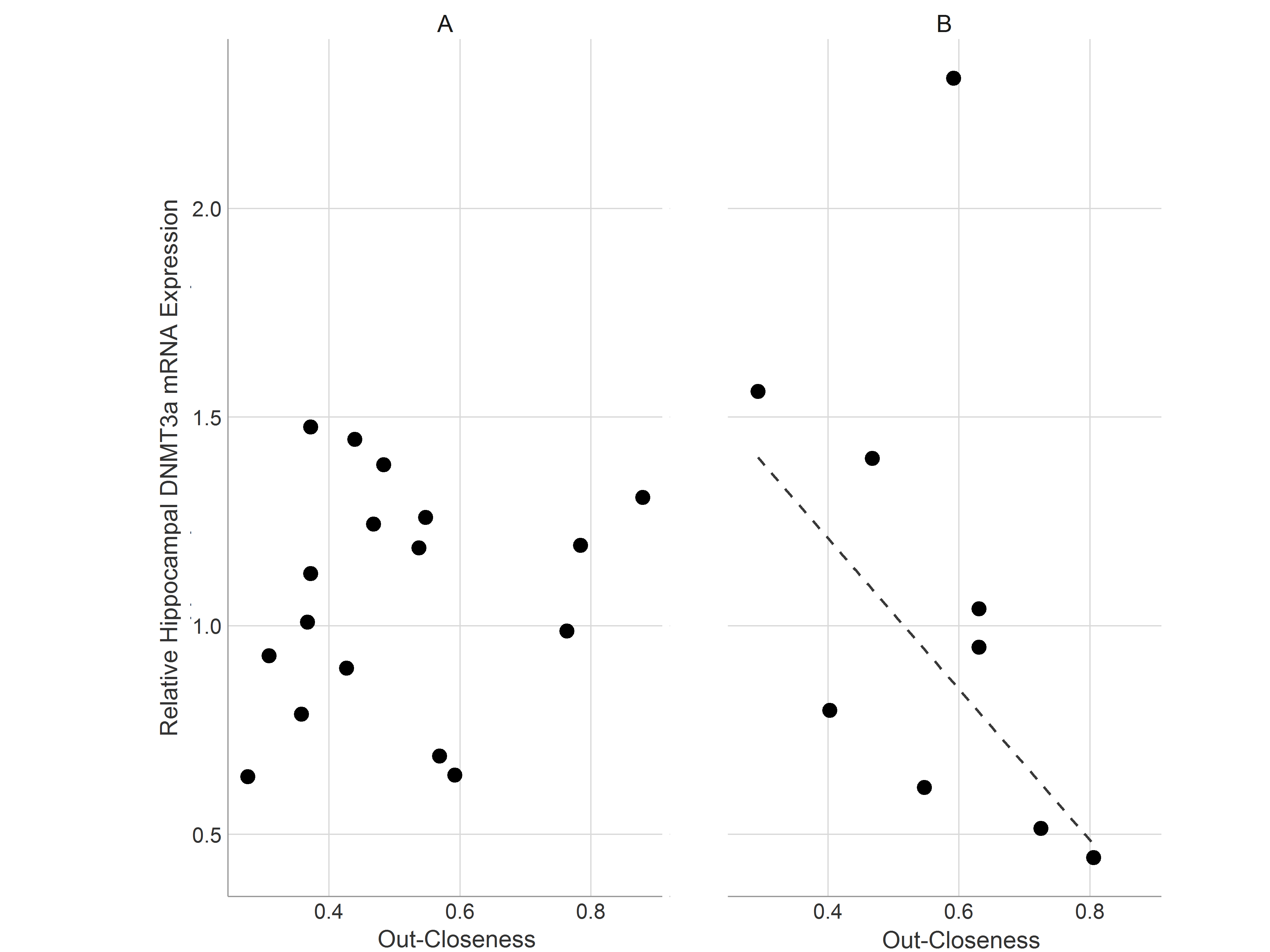


**Supplemental Figure 7.** mPOA **(A)** DNMT1 and **(B)** DNMT3a expression are not associated with Out-Closeness. Each point represents one individual with color representing the network community of that individual (orange – community A, dark gray – community B, light gray – other).


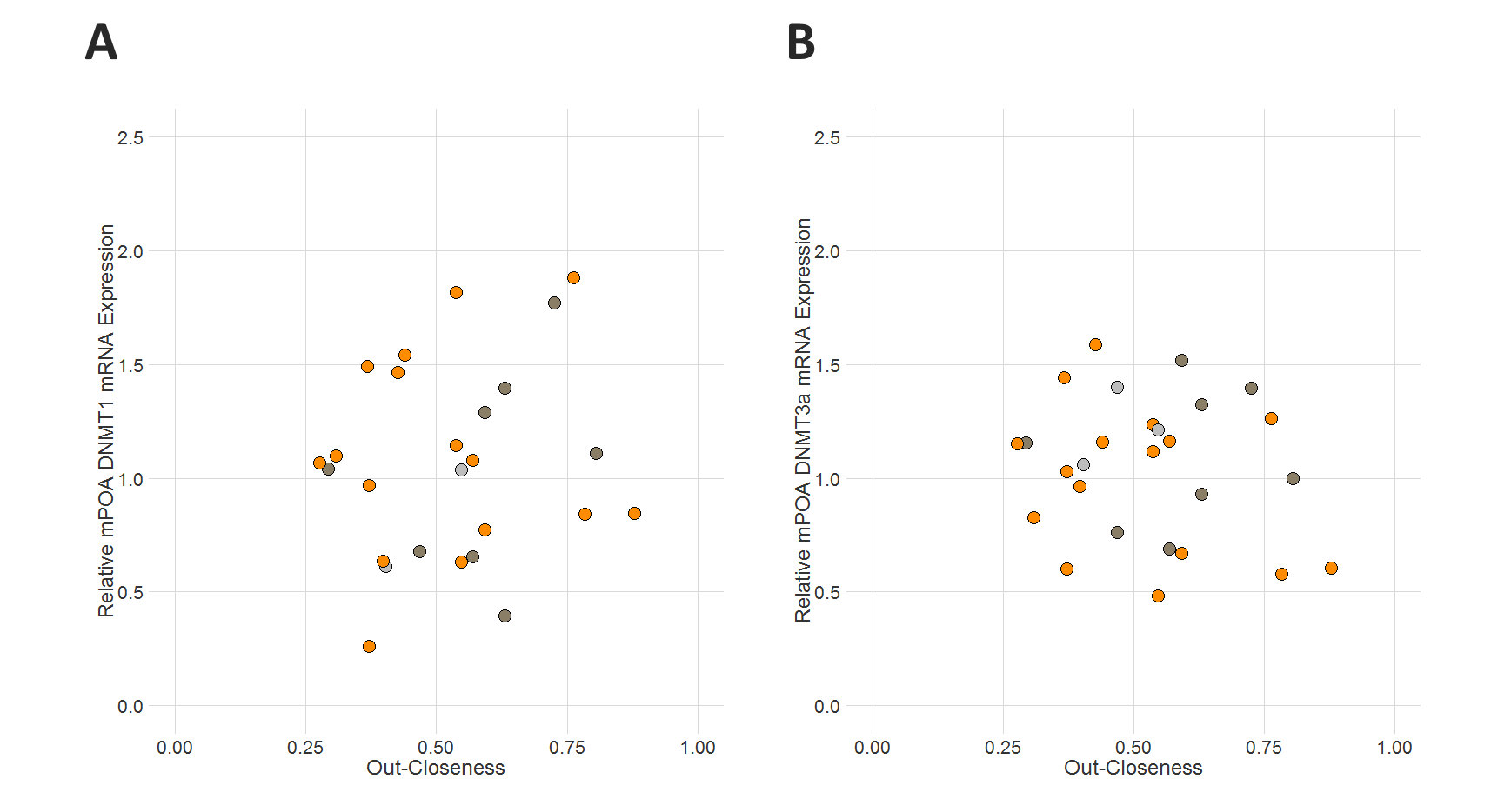

Supplement: Supplementary file 1 [file Table_1.DOCX]
